# The Natural Variation of a Neural Code

**DOI:** 10.1371/journal.pone.0033149

**Published:** 2012-03-12

**Authors:** Yoav Kfir, Ittai Renan, Elad Schneidman, Ronen Segev

**Affiliations:** 1 Department of Biomedical Engineering, Ben-Gurion University of the Negev, Beer-Sheva, Israel; 2 The Zlotowski Center for Neuroscience, Ben-Gurion University of the Negev, Beer-Sheva, Israel; 3 Department of Zoology, George S. Wise Faculty of Life Sciences, Tel Aviv University, Tel Aviv, Israel; 4 Department of Neurobiology, Weizmann Institute of Science, Rehovot, Israel; 5 Department of Life Sciences, Ben-Gurion University of the Negev, Beer-Sheva, Israel; Imperial College London, United Kingdom

## Abstract

The way information is represented by sequences of action potentials of spiking neurons is determined by the input each neuron receives, but also by its biophysics, and the specifics of the circuit in which it is embedded. Even the “code” of identified neurons can vary considerably from individual to individual. Here we compared the neural codes of the identified H1 neuron in the visual systems of two families of flies, blow flies and flesh flies, and explored the effect of the sensory environment that the flies were exposed to during development on the H1 code. We found that the two families differed considerably in the temporal structure of the code, its content and energetic efficiency, as well as the temporal delay of neural response. The differences in the environmental conditions during the flies' development had no significant effect. Our results may thus reflect an instance of a family-specific design of the neural code. They may also suggest that individual variability in information processing by this specific neuron, in terms of both form and content, is regulated genetically.

## Introduction

The nervous system relies on a nearly universal “alphabet”: most neurons, in different brain modules and in many different species, use sequences of electrical spikes to represent and transmit information [1]. However, the structure of this “neural code” – i.e. the way information is carried by spike trains and how it can be read – can differ even between brain areas in the same animal. The similarity in anatomical and functional organization of the nervous system in different individuals reflects the universal properties of brain design and function. However, it is unclear how different the corresponding brain modules of two animals are, or how they differ in terms of the computations they perform.

Behavioral differences between individuals are often attributed to genetic variations between species or even between individuals, which are likely to be reflected in the neural circuit architecture, synaptic connection patterns, etc. [2], [3]. Sensory experience also has a profound impact on the structure and performance of the nervous system [4]. Here we aimed at a detailed quantitative analysis of the differences between individuals at the level of their neural codes. The putative design of the neural code as regards efficiency in terms of energy [5], [6], information content [7], [8], or predictive value [9] has been explored in different neural systems. We expect that differences (or similarities) that are informative about the “conserved” and idiosyncratic parts of the neural code could have a functional relation to behavioral differences between individuals, species, or families. To conduct such analyses, we need a quantitative framework in which to explore neural code variability, which could then be related to behavioral variability.

We used the identified H1 neuron in the fly visual system to study the individual nature of the neural code. H1 is a motion sensitive neuron that processes wide field visual motion, and is part of a network that is responsible for stabilizing yaw optomotor responses [10], [11]. In each brain hemisphere there is one H1 neuron that responds to horizontal back to front motion and is inhibited by backwards motion; each one of the neurons projects to the contra-lateral hemisphere of the fly's brain. This neuron has been widely used in the study of various properties of the neural code, including the reliability of its response, information content, the temporal structure of its code and its adaptation properties [7], [12]–[22], and its sensitivity to the fly's activity state [23], [24]. Within the same species, the H1 code can demonstrate both high variability, and some universal properties [17]. It is also well known that sensory experience has an extensive effect on the development of the fly's peripheral visual system [25], [26], although, as shown by [25], rearing flies in a dark environment did not affect the response of H1 to local visual stimuli in a small region of the visual field. Here we focused on differences between two fly families, and explored the effect of sensory experience on the structure of the neural code at the level of individuals.

## Results

We analyzed the structure and content of the spiking patterns of the identified H1 neuron in 24 flies from two families - six blow flies, and 18 flesh flies – responding to wide field motion stimuli. H1 is a directional selective spiking neuron in the lobula plate, which is selective for wide field horizontal motion in front of the fly's eye. We identified the H1 neuron according to its response properties; namely, directional selectivity and spiking response to wide field horizontal motion in front of the fly's contra-lateral eye (see methods).

**Figure 1:**
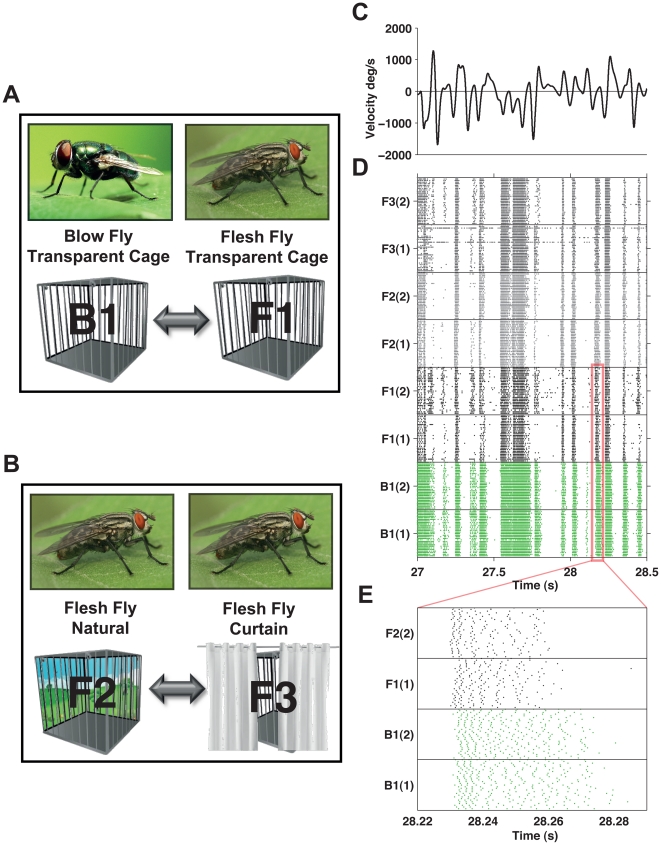
An overview of the experimental setup and H1 neuron responses to dynamic stimuli. (**A**) Illustration of the first part of the experiment: comparing the neural activity of different fly families, *Calliphoridae* (blow flies, group B1) and *Sarcophagidae* (flesh fly group F1). (**B**) Illustration of the second part of the experiment: comparing the neural activity of flies from the same family (flesh flies) that were exposed to different environmental conditions, natural surroundings (group F2) or covered by a white curtain (group F3). (**C**) Stimulus, horizontal velocity of moving bars. Negative values correspond to motion in the preferred direction of the neuron. (**D**) Raster plots of two flies from each group. Each line represents one repetition of the response to the stimulus shown in C, with each dot representing the occurrence of a spike. (**E**) Magnification of a short section of the response of flies from groups B1 and F1. Note that the disparity between the two families is clearly apparent in response times and firing rates.

To compare the neural code of H1 in the two families we bred blow flies (Fig. *1A*, group B1, n = 6) under similar visual conditions as a group of flesh flies (group F1, n = 6). To estimate the effect of the environment on the neural code we further studied two other groups of flesh flies (six individuals each), which were bred under different visual environments: one group was bred outdoors in a transparent cage (Fig. *1B*, group F2), and the other (F3) was bred outside but in an opaque cage, which exposed the flies to similar natural light intensities, but no details of the natural environment.

After the flies matured, we recorded extracellularly the activity of H1 in response to wide field stimulation by a pattern of random horizontal motion of vertical bars (Fig. *1C*). Figure 1D presents examples of the spiking patterns of the H1 neurons in eight flies (two from each group), with respect to the same movie segment. H1 responds with high firing rates when the horizontal motion is in the preferred direction of the neuron, whereas motion in the opposite direction results in almost complete silence [11]. Differences between the two families were already clear from their average firing rates: flesh flies had 59±4 *spikes/s* (n = 6) and blowflies had 86±5 *spikes/s* (n = 6). The families also clearly differed in their response latencies (Fig. 1E): blow flies were significantly delayed compared to flesh flies (∼2 ms difference) and were also much more variable (the response of blow flies appears less repetitive, which we will later quantify as higher entropy values). However, importantly, the differences between families were much more significant and intricate than firing rate or delay alone.

### Different families use different neural vocabularies and code

To systematically compare the neural code of the different flies from the different groups, we discretized the activity patterns of their H1 neurons into small bins of Δt = 2 milliseconds, denoting spiking in a given bin as ‘1’ and silence as ‘0’. Using this standard binary representation [7], we characterized the vocabulary of binary ‘words’ that each of the flies used to encode the visual stimulus. This was done using the probability distribution over the words that the *i^th^* fly used during the whole experiment, 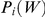, and the distribution of words that the *i^th^* fly used to encode the same stimulus segment, *s*, denoted by 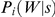. We estimated the conditional distribution from repeated presentations of the same stimulus.

**Figure 2:**
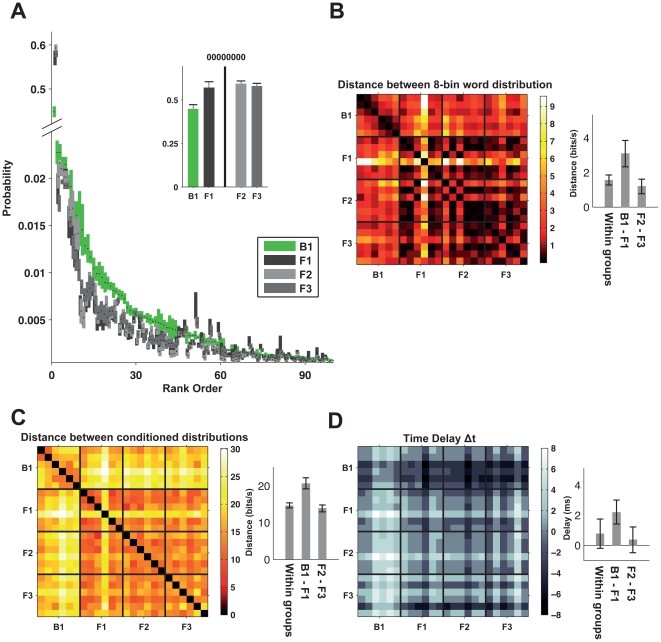
Word distributions of flies and a comparison between codewords of different fly families. (**A**) Mean and standard error values of the distribution of 8-letter words in the four groups. Words are arranged in decreasing rank order of the probability of words in the response of blow flies (note that the whole range of 256 possible words is not shown). Inset – mean and standard error of the probability of ‘00000000’ which was the most commonly used word. (**B**) Matrix of the Jensen Shannon distances between 8-letter word distributions of each pair of flies. Bars show mean values+SEM of three clusters: distance within each of the four groups, between fly families (groups B1 and F1) and flies from different environmental conditions (groups F2 and F3). (**C**) Matrix of the distances between the stimulus conditioned word distribution of each pair of flies, averaged across the stimulus presentation. Bars show mean+SEM values of the same clusters as in B. (**D**) The distance between each two flies was minimized by finding the time shift between their responses that resulted in the minimal value (see Methods). Matrix displays the time shift in milliseconds between the responses of each pair of flies that minimized the distance. Bars show mean+SEM values of same clusters as in **B**. A similar analysis using 12-letter words produced qualitatively similar results.

Figure 2A shows the group-averaged probability distribution of 8-‘letter’ long words (a total duration of 16 ms) used by the blow flies 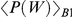 (green, 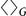 denotes averaging over group *G*), and the probability of the same words for the different flesh fly groups 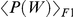, 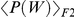 and 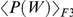 (different shades of gray). To avoid bias from the specific choice of code word length, our analysis focused on the similarity or difference in codewords, and their information content, per unit time. We therefore compared the flies using codewords of length 1,2,4,8 and 16, and normalized by the word length. We focus here on 8-letter words as these are long enough to allow the temporal structure of codewords to be studied [26], but still enable us to reliably sample the distribution of the words [27]. It is easy to distinguish between fly families even based on single words: the most common word of all the flies was ‘00000000’ (Fig. *2A*, inset), but blow flies used this word about 40% of the time compared to almost 60% of the time in flesh flies.

We quantified the differences between the “vocabulary” of spiking patterns of the two families, by the dissimilarity of the distributions of code words they used. The Jensen-Shannon (JS) divergence [28] between two distributions, P and Q, is a symmetrized, and bounded extension of the commonly used Kullback-Leibler (KL) divergence between probability distributions [29], and measures in bits how easy it is to tell the distributions apart (Fig. *2B*). Recall that the KL divergence between P and Q is given by
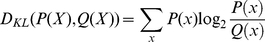
(1)The JS divergence between P and Q, ranges from ‘0’ which indicates that the two distributions are identical, and ‘1’ when they have no overlap.
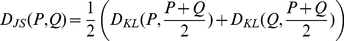
(2)We then quantified the dissimilarity between the distributions of words *fly i* used in response to the whole movie, and those that *fly j* used:
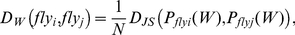
(3)where N is the length of word W. We found that the code words of the two families were 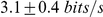 apart, whereas only 1 bit is needed to tell them apart with certainty. Thus, given a neural response segment of a fly, and knowing the total distribution of words that the flies use, about 300 ms are needed to identify to which of the two families it belongs. The differences between the different groups of flesh flies were less pronounced, at 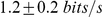, which was similar to the difference between individual flies within each of the flesh fly groups (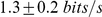).

To quantify the difference in the way the flies use their vocabularies to convey information about the stimulus, we compared the distribution of codewords that each of the flies used to encode the same stimulus segments over repeated presentations of a 40 second long stimulus. Thus, for each stimulus segment, *s*, we estimated the difference between the local distributions of codewords that flies *i* and *j* used, 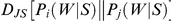 and averaged it across all stimulus segments. Since exactly the same stimulus was repeated in every trial, we can average over time instead of stimulus segments.
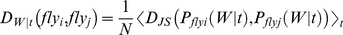
(4)The average of all the pairwise distances between blow flies and flesh flies was 20.7±0.7 *bits/s* (B1 vs. F1, Fig. *2C*), whereas the typical difference between conspecifics was 14.0±0.4 *bits/s* (F2 vs. F3). Importantly, we quantified the difference between the neural codes of the flies solely from the differences in the instantaneous firing rates, by using 1 bin words. We found that the average distance between families was 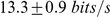; hence the temporal structure of the words increased the difference between the codes by more than 35%. This again reflects that the difference between these families goes far beyond differences in firing rates.

To verify that the differences between the stimulus dependent word distributions of the two families were not only attributable to the different temporal response delays shown in figure *1E*, for each pair of flies we calculated the time shift between the responses of the flies, Δ*T*, that minimized the Jensen-Shannon distance between them.
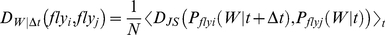
(5)We found that the distance between the families was slightly reduced to 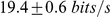 and between the flesh fly groups to 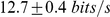. The values of the time shifts (Fig. *2D*) that resulted in the minimal distance between flies in the blow fly and flesh fly groups was ∼2 ms (significantly different from zero, P<0.01, permutation test), indicating that flesh flies responded faster than blow flies to the stimulus. Among the flesh fly groups the timing differences were not significantly different from zero, similar to the average time shift that minimized the distances between fly pairs within each group.

### Experiencing different visual environments during early development after pupation showed no significant effect on the neural code

We found no effect of the visual environment that the flies were exposed to from pupation on the neural ‘vocabularies’ of the different flesh fly groups. This is consistent with Karmeier *et al.*, who saw no effect of breeding flies in the dark on their receptive field properties. Moreover, we found no differences in the vocabulary they used to encode the stimulus. Fig. 2 B and C show that the codes of the flesh flies that were exposed to different visual environments were as different as the variability among conspecifics from the same habitat (Fig. *2B,C* inset), both in terms of firing rate and temporal structure of the code.

### The neural code of males and females was indistinguishable

We repeated the same analysis for males and females in our flies. We found no difference between the sexes, as the average distance was similar to the distances found within each gender (within the male group and within the female group, Fig. *S1*). Moreover, the distance we observed between the sexes was similar to the distance between individual flies that was discussed above. It should be noted that since we could not identify with certainty the species of the female flesh flies our result here is limited in scope.

### Codeword differences between fly families

**Figure 3:**
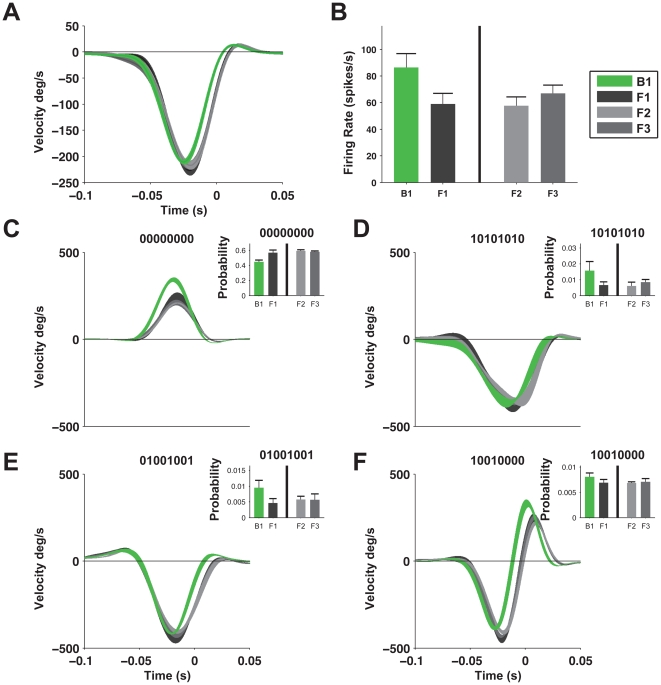
Neural responses of the two fly families convey different information about the stimulus. (**A**) STA, spike-triggered average, the average stimulus that preceded the occurrence of a single spike, averaged in the four groups. Line width represents standard error of the mean. The STA of the blow fly group was significantly different from the STA of all flesh fly groups. (**B**) Mean firing rates and standard error of the four groups; again the blow fly exhibited significantly higher rates. (**C–F**) Four examples of word triggered averages (WTAs); the average stimulus that preceded the occurrence of each specific 8-letter word. Inset - corresponding probabilities that each word will appear in the response of each group, mean+SEM.

To characterize the differences in the information carried by the neural codes of the different species, we mapped the relation between stimuli and the neural responses that the flies used to encode them. Figure 3A shows the average stimulus preceding a single spike (known as the spike triggered average, or STA) of each of the groups. Similar to what we found when comparing the codewords themselves, the STA of blow flies differed from those of the flesh flies (all groups) in width and time of the peak, and was consistent with the time shift that minimized the distance between the responses in figure 2D. Again, we found no differences between the different groups of flesh flies.

To explore the role of combinatorial coding patterns in the two species, we estimated the word-triggered average (WTA), or the average stimulus that preceded each of the 8-letter words in the vocabulary [30]. Figures 3C–F depict four examples of the WTA of frequent words, and the corresponding probability of these words in the different groups. Specifically, we found words that have different WTAs between the species and different probabilities of use by each of the species (e.g. the word ‘00000000’, Fig. *3C*). We also found words (e.g. ‘10101010’, Fig. *3D*) that had similar WTAs for all flies but with different probabilities for the two species, and words that had different WTAs but appeared with similar probabilities. In all cases, we did not find any significant differences between the three groups of flesh flies.

### Blow flies' H1 carries more information than flesh flies using a noisier code

To measure the differences in content of the neural codes of the flies, we assessed how much information they carried about the same stimulus [7], [12]. The mutual information between the responses *(W)* and the stimuli *(S)* is given by the difference between two terms: the total entropy of the neural vocabulary [8], [31].
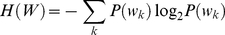
(6)and the entropy of the “noise” in the neural response,
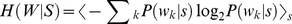
(7)which together gives
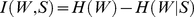
(8)The entropy of this distribution *H(W)* measures the richness of the vocabulary of the cell and is a bound on the information coding capacity of the cell. The noise entropy of the response, *H(W|S)*, to each stimulus segment was estimated from repeated presentations of the stimulus. The average noise entropy over stimulus segments was replaced by averaging over stimulus time [27] such that the mutual information between the stimulus and response is given by:
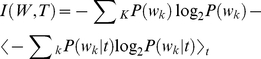
(9)We estimated information rates by extrapolating the information estimate for finite words to infinite word length and data size as in Strong *et al.* [27]. The average information carried per spike was estimated by dividing the amount of information carried by the neuron per second by the number of spikes that the flies used per second.

**Figure 4:**
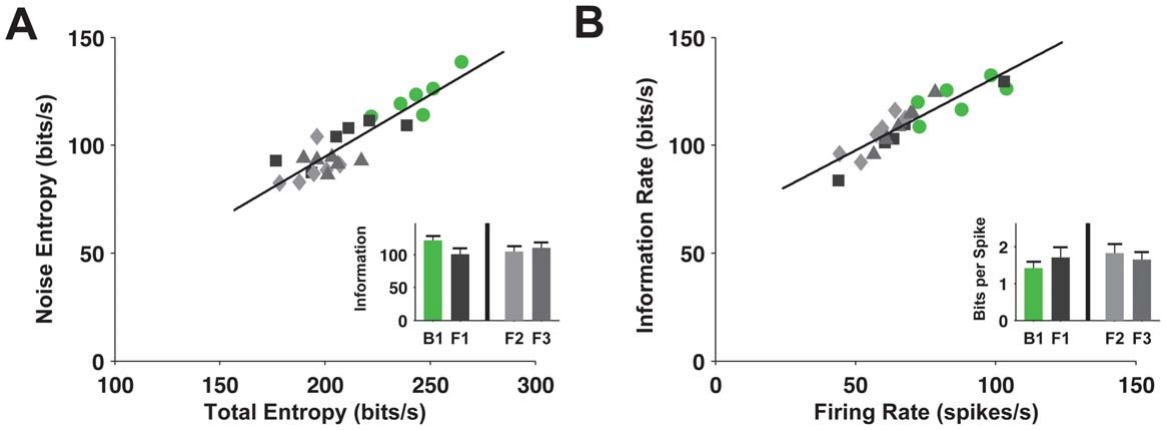
Blow flies convey more information but less efficiently. (**A**) Noise entropy rate vs. of entropy rate. Each data point represents an individual fly, colors are according to groups. Inset: mean and standard error of mutual information rates. (**B**) Information rate vs. firing rate; again each data point represents an individual fly, colors are according to groups. Inset: mean and standard error of information per spike.

Figure 4A shows the noise entropy rate as a function of the total entropy rate for all the flies. We found that blowflies had a more diverse codebook than the three groups of flesh flies (higher total entropy), but that this code was also noisier (higher noise entropy). There were no significant differences between the total entropy and noise entropy for the different groups of flesh flies.

### Flesh flies use a more efficient neural code

By estimating the information carried by the flies per unit time, we found that the blowflies encoded 20% more information about the stimulus than the flesh flies (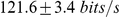, vs. 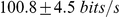, P<0.025). To convey the additional information blowflies used firing rates that were about 45% higher than flesh flies 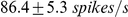, vs. 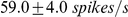, P<0.034). Despite the higher information rates conveyed by blowflies, the average information carried by each spike was 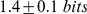, whereas the information per spike carried by flesh flies was higher, 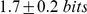 (Fig. *4B* inset), suggesting a higher energetic efficiency of the flesh fly code (P = 0.034, permutation test).

## Discussion

We showed that the neural code of an identified neuron that encodes similar visual properties in two fly families differs considerably. The H1 neuron in the blow fly conveys more information about the stimulus than in the flesh fly, although blow flies use much higher firing rates than flesh flies presented with the same visual stimuli. The energetic efficiency of the code also differs between the fly families, as blow flies carry less information with each spike. For all the comparisons that exhibited differences between families, there were no significant differences between groups of flies from the same family (flesh flies) that were exposed to different visual environments. We concluded that manipulation of the visual environment soon after pupation does not affect the firing rate of this specific neuron, or more complex properties of the neural code that displayed differences between the two fly families.

There was no effect of the visual environment experienced by the flies on the code of their H1 neuron. Our results are in line with the study of Karmeier *et al.* [25] who showed that rearing flies in darkness does not affect the firing rate of the H1 neuron in response to local visual stimulation compared to normal surroundings. One possible explanation is that flies need to fly almost immediately after they hatch from the cocoon, and thus the optomotor system, and H1 in particular, are genetically hard-wired although some response properties, as response latency, vary with the age of the fly [32]. Moreover, since H1 is highly adaptive [19], [21], [33], flies could rely on its dynamic range to allow for matching the coding properties to the environment. If so, adaptation of the code to new environment statistics would occur only over generations due to evolutionary pressure.

Niven *et al.* [34] examined the relation between information content and energy consumption in the responses of photoreceptors of flies of different species. They showed that the relationship between information rate and energy follows the law of diminishing returns: increasing encoded information rates requires more energy per bit. Assuming that the H1 neuron of both fly families uses the same amount of energy to produce a spike, our results show that a similar rule exists as well at higher levels of the fly's visual system. Blow flies use 45%±7% more spikes than flesh flies to convey information about the stimulus, which requires investing higher amounts of energy compared to flesh flies. However, the additional amount of information does not increase in a similar fashion, and the response contained only 20%±5% more bits per second. Interestingly, Borst and Haag [13] demonstrated a similar sub-linear increase in information rate of the H1 neuron by manipulating the stimulus to increase the cell's firing rate in the same fly.

It is not immediately clear why H1 neurons of blow flies carry more information than those of flesh flies or why they use 45% more spikes to encode wide-field horizontal motion. As H1 is part of the optomotor system, and the information it conveys is used for determining the flight pattern of the fly, the disparity between the neural codes may be optimized for the species' flight properties in terms of flight velocities, optic flow and turning speeds. The significant differences we have found in the code and processing of information between the two families suggest a further exploration of the relation of the specifics of the neural code and the behavioral phenotypes. Identified neurons, like H1, could be used for a comparative analysis over several species, linking behavior and the neural code, by using the framework presented here to quantify coding differences.

We recorded H1 activity from immobilized flies, which minimizes artifacts that result from self-motion and thus provide a clear view of the differences in the encoding schemes of the two families. Since the activity of H1 depends on the behavioral state of the fly [23], [24], a comparison of the codes of the same neuron under more natural fly-like conditions, might reveal other differences between families or species, and show possible links between the properties of the neural code and behavior.

The biophysical sources of the differences we observed in the structure and content of the code across families – either at the level of H1 itself, or in the synaptic inputs it receives are promising directions for future work.

## Methods

### Experimental Models

We compared the activity of two fly families: the green bottled blow fly *Calliphoridae*, *Chrysomya albiceps*, and *Sarcophagidae* flesh flies. The male flesh flies belonged to two species, *Aegyptica* and *Argyrostoma*. The females of the flesh fly family could not be identified at the level of the species. All the flies are relatively the same size, and were caught outdoors in Beer-Sheva, Israel. To obtain flies, meat was placed outdoors. Several days later, maggots, the fly larval phase, were collected and placed in transparent plastic cages. After about two to three weeks from pupation, adult flies hatched. We began by comparing flies from two different families. Six flies from each family, blow flies and flesh flies, were kept under identical conditions inside the lab (Fig. *1A*). We further compared flies from the same family that experienced different environmental conditions. Twelve flesh flies were separated after pupation into two groups and kept in cages placed outdoors among the bushes. One group was placed in a transparent cage and viewed a natural scene of vegetation. The second group was kept nearby under similar conditions but a white curtain enclosed the cage. Other than the passage of light, the curtain blocked the total view of the natural environment around the flies (Fig. *1B*). All flies in each group came from the same pool; hence, although we could not identify the females to the level of the species, they are most likely similar to the males. Flies from all groups were 5–12 days old when we recorded their H1 neuron activity, after experiencing these environmental conditions for at least five days. All groups of flies contained both males and females flies with at least a third of each sex in every group.

This study was conducted according to animal welfare regulations for invertebrates in Israel; no specific permits were required.

### Electrophysiology

We used a standard extracellular method [35] to record the activity of the H1 neuron. The fly was anesthetized by keeping it at a low temperature for few minutes until it ceased moving. It was then immobilized in wax and its head fixed to look downwards by a tiny drop of adhesive. A small hole was made through the cuticle in the back of its right eye and reference silver wire coated with silver chloride and a tungsten microelectrode (1 MΩ, FHC, USA) was inserted to capture extracellular signals generated by the H1 neuron. In both fly families we identified the neuron by a spiking response to wide-field motion towards the center of the field of view in front of the left (contra-lateral) eye and inhibition due to motion in the opposite direction. To our knowledge, we are the first to identify the H1 neuron in flesh flies according to these criteria, and this neuron has yet to be identified by other common techniques. Thus, we cannot exclude that we recorded from a different neuron that share the exact same properties used to identify H1. The signal was filtered and amplified by a DAM-50 amplifier (WPI, USA) digitized at 10 kSamples/s by NI-6071 A/D converter (National Instruments, Israel). Recording procedures for H1 activity of all flies were carried out at constant room temperature (22°–24°), similar to the average outside temperature in Beer Sheva, Israel at time of the recording. Stimulus display and data recordings were carried out by a single dedicated LABVIEW program. Data analysis was done using MATLAB. A dedicated off-line threshold-crossing algorithm was used to detect spikes.

### Visual Stimulation

We used a directed beam monitor, Tektronix 608, to present motion stimuli to the flies with an average frame rate of over 3000 frames/s. The monitor was covered with an opaque material that had a 80 mm round shaped hole. All flies were placed 120 mm away from the monitor in the same orientation that resulted in stimulating the center of the visual field of the left eye. Each fly was presented with one hour of a non-repeating movie, followed by three hours of repeated stimuli composed of 256 repetitions of an identical 40-second movie. In both cases, the stimulus was a moving pattern of vertical bars with random widths (On average, the total coverage of illuminated bars was 60% of the screen). We maintained constant luminance and constant contrast (contrast level – 0.92) throughout the whole movie. The bar pattern executed a horizontal zero mean random walk, with a normally distributed sequence of step lengths that were interpolated to produce a smooth appearance of movement (μ = 0 deg/s, δ = 470 deg/s).

### Statistical significance measure

Statistical significance of the similarity between groups of flies (firing rates, codeword entropy, information, JS divergence) was estimated by comparison to the values between many randomly assigned groups of flies (when each fly was randomly assigned to one of the groups, regardless of its species or background). All significance values were higher than P<0.01 (or as otherwise noted in the text).

## Supporting Information

Figure S1 **A comparison between codewords of different sexes.** Matrix of the Jensen Shannon distances between 8-letter word distributions of each pair of flies, similar to figure 2B, but clustered according to sex. Bars show mean values+SEM of the distances within each of the two clusters.
(TIF)

## References

[1] Adrian ED (1926). The impulses produced by sensory nerve-endings: Part I. The Journal of physiology.

[2] Goaillard J, Taylor A, Schulz D, Marder E (2009). Functional consequences of animal-to-animal variation in circuit parameters.. Nature neuroscience.

[3] Thompson P, Cannon T, Narr K, van Erp T, Poutanen V (2001). Genetic influences on brain structure.. Nature neuroscience.

[4] Hubel D, Wiesel T (1970). The period of susceptibility to the physiological effects of unilateral eye closure in kittens.. The Journal of physiology.

[5] Levy WB, Baxter RA (1996). Energy efficient neural codes.. Neural Computation.

[6] Laughlin SB (2001). Energy as a constraint on the coding and processing of sensory information.. Current Opinion in Neurobiology.

[7] Rieke F, Warland D, van Steveninck RR, Bialek W (1997). Spikes: Exploring the neural code.

[8] Borst A, Theunissen FE (1999). Information theory and neural coding.. Nature neuroscience.

[9] Srinivasan M, Laughlin S, Dubs A (1982). Predictive coding: a fresh view of inhibition in the retina.. Proceedings of the Royal Society of London Series B Biological Sciences.

[10] Hausen K (1984). The lobula-complex of the fly: structure, function and significance in visual behaviour.. Photoreception and vision in invertebrates.

[11] Eckert H (1980). Functional properties of the H1-neurone in the third optic ganglion of the blowfly, Phaenicia.. Journal of Comparative Physiology A: Neuroethology, Sensory, Neural, and Behavioral Physiology.

[12] Bialek W, Rieke F, de Ruyter Van Steveninck RR, Warland D (1991). Reading a Neural Code.. Science.

[13] Borst A, Haag J (2001). Effects of mean firing on neural information rate.. Journal of Computational Neuroscience.

[14] Borst A (2003). Noise, not stimulus entropy, determines neural information rate.. Journal of Computational Neuroscience.

[15] Haag J, Borst A (1997). Encoding of visual motion information and reliability in spiking and graded potential neurons.. The Journal of neuroscience.

[16] Nemenman I, Lewen GD, Bialek W, van Steveninck RRR (2008). Neural Coding of Natural Stimuli: Information at Sub-Millisecond Resolution.. PLoS Computational Biology.

[17] Schneidman E, Brenner N, Tishby N, de Ruyter van Steveninck RR, Bialek W (2001). Universality and individuality in a neural code.. Advances in neural information processing systems.

[18] Grewe J, Kretzberg J, Warzecha AK, Egelhaaf M (2003). Impact of photon noise on the reliability of a motion-sensitive neuron in the fly's visual system.. The Journal of neuroscience.

[19] Maddess T, Laughlin SB (1985). Adaptation of the motion-sensitive neuron H1 is generated locally and governed by contrast frequency.. Proceedings of the Royal Society of London Series B Biological Sciences.

[20] Grewe J, Matos N, Egelhaaf M, Warzecha AK (2006). Implications of functionally different synaptic inputs for neuronal gain and computational properties of fly visual interneurons.. Journal of neurophysiology.

[21] Warzecha AK, Egelhaaf M (1999). Variability in spike trains during constant and dynamic stimulation.. Science.

[22] Kurtz R, Egelhaaf M, Meyer HG, Kern R (2009). Adaptation accentuates responses of fly motion-sensitive visual neurons to sudden stimulus changes.. Proceedings of the Royal Society B: Biological Sciences.

[23] Rosner R, Egelhaaf M, Warzecha AK (2010). Behavioural state affects motion-sensitive neurones in the fly visual system.. Journal of Experimental Biology.

[24] Jung SN, Borst A, Haag J (2011). Flight Activity Alters Velocity Tuning of Fly Motion-Sensitive Neurons.. The Journal of neuroscience.

[25] Karmeier K, Tabor R, Egelhaaf M, Krapp HG (2001). Early visual experience and the receptive-field organization of optic flow processing interneurons in the fly motion pathway.. Visual neuroscience.

[26] Brenner N, Bialek W, de Ruyter van Steveninck R (2000). Adaptive rescaling maximizes information transmission.. Neuron.

[27] Strong SP, Koberle R, De Ruyter Van Steveninck RR, Bialek W (1998). Entropy and Information in Neural Spike Trains.. Physical review letters.

[28] Lin J (1991). Divergence measures based on the Shannon entropy.. IEEE Transactions on Information theory.

[29] Cover TM, Thomas JA (2004).

[30] Van Steveninck RDR, Bialek W (1988). Real-time performance of a movement-sensitive neuron in the blowfly visual system: coding and information transfer in short spike sequences.. Proceedings of the Royal Society of London Series B, Biological Sciences.

[31] Shannon C (1948). A mathematical theory of communication.. Bell System Technical Journal.

[32] Warzecha AK, Egelhaaf M (2000). Response latency of a motion-sensitive neuron in the fly visual system: dependence on stimulus parameters and physiological conditions.. Vision research.

[33] Fairhall AL, Lewen GD, Bialek W, de Ruyter van Steveninck RR (2001). Efficiency and ambiguity in an adaptive neural code.. Nature.

[34] Niven JE, Anderson JC, Laughlin SB (2007). Fly photoreceptors demonstrate energy-information trade-offs in neural coding.. PLoS Biol.

[35] Warzecha AK, Egelhaaf M (1997). How Reliably Does a Neuron in the Visual Motion Pathway of fhe Fly Encode Behaviourally Relevant Information?. European Journal of Neuroscience.

