# Supplementary figures and images for: The Natural Variation of a Neural Code
Source: PLoS One. 2012 Mar 12;7(3):e33149. doi: 10.1371/journal.pone.0033149 (PMC3299747; doi:10.1371/journal.pone.0033149)

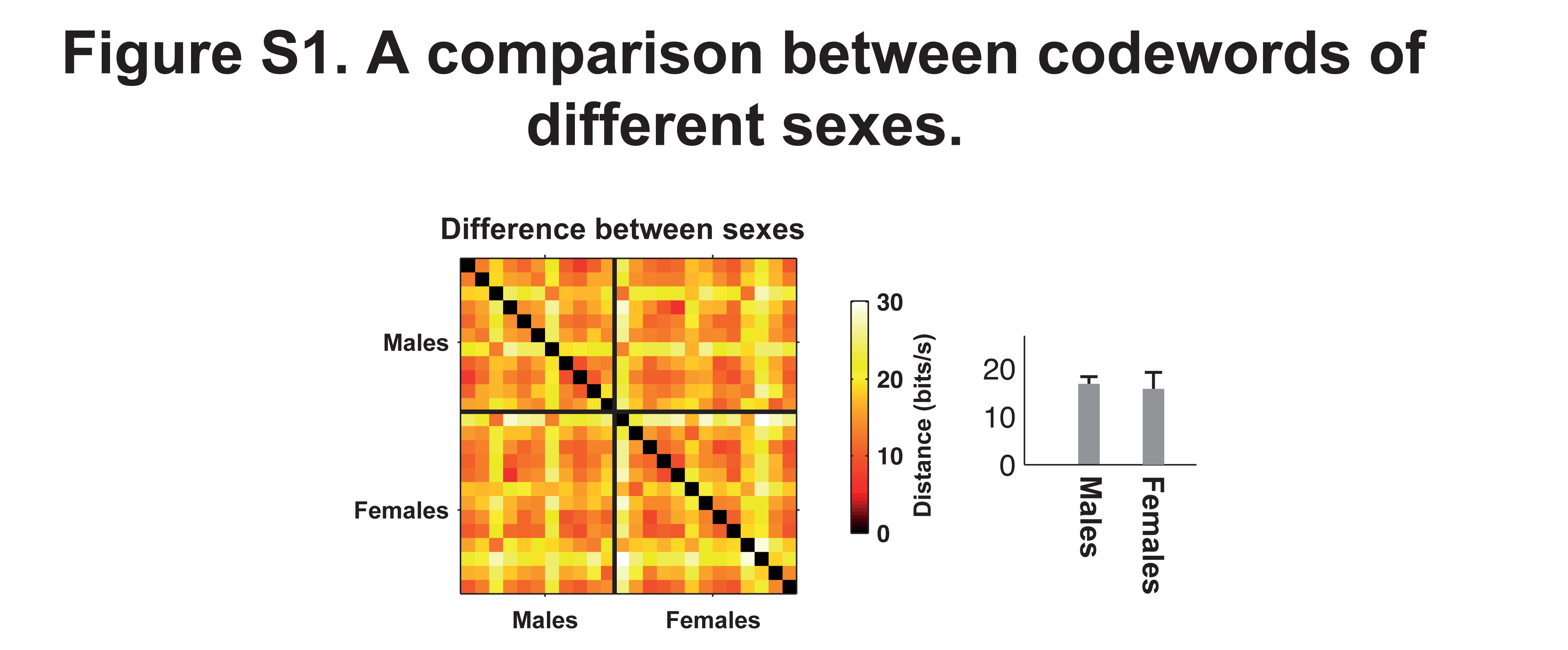

Supplement: Figure S1 — A comparison between codewords of different sexes. Matrix of the Jensen Shannon distances between 8-letter word distributions of each pair of flies, similar to figure 2B, but clustered according to sex. Bars show mean values+SEM of the distances within each of the two clusters. (TIF) [file pone.0033149.s001.tif]
